# Giant Frontal Paranasal Mucocele: Case Report and Review of the Literature

**DOI:** 10.5334/jbsr.2117

**Published:** 2020-09-11

**Authors:** Frederik Bosmans, Filip Vanhoenacker

**Affiliations:** 1 AZ Sint-Maarten, BE; 2 AZ Sint-Maarten and University (Hospital) Antwerp/Ghent, BE

**Keywords:** giant mucocele, paranasal sinuses, CT, MRI

## Abstract

**Teaching point:** Giant mucocele is a rare expansile lesion that may mimic other locally aggressive lesions of the cranial vault.

Giant frontal mucoceles with massive osteolytic destruction mimicking an aggressive lesion are rare compared to smaller mucoceles. This article reports a giant mucocele of the frontal sinus and reviews the literature. Important imaging clues pointing toward the diagnosis of a mucocele on computed tomography (CT) and magnetic resonance imaging (MRI) are a well-defined expansile mass, an intimate relationship with the frontal sinus, subtle peripheral rim enhancement, and slow progression on serial imaging. The density on CT and signal on MRI may vary along with the lesion content. The potential role of diffusion-weighted imaging should be elaborated in future reports.

## Introduction

Giant mucoceles of the frontal sinus are rare clinical entities, and only a few cases are published in the current literature [1,2,3,4,5,6]. This case report will briefly summarize the etiopathogenesis and clinical presentation of sinus mucoceles. The imaging findings on computed tomography (CT) and magnetic resonance imaging (MRI) are discussed in detail, along with a review of the relevant literature.

## Case Report

**Figure 1:**
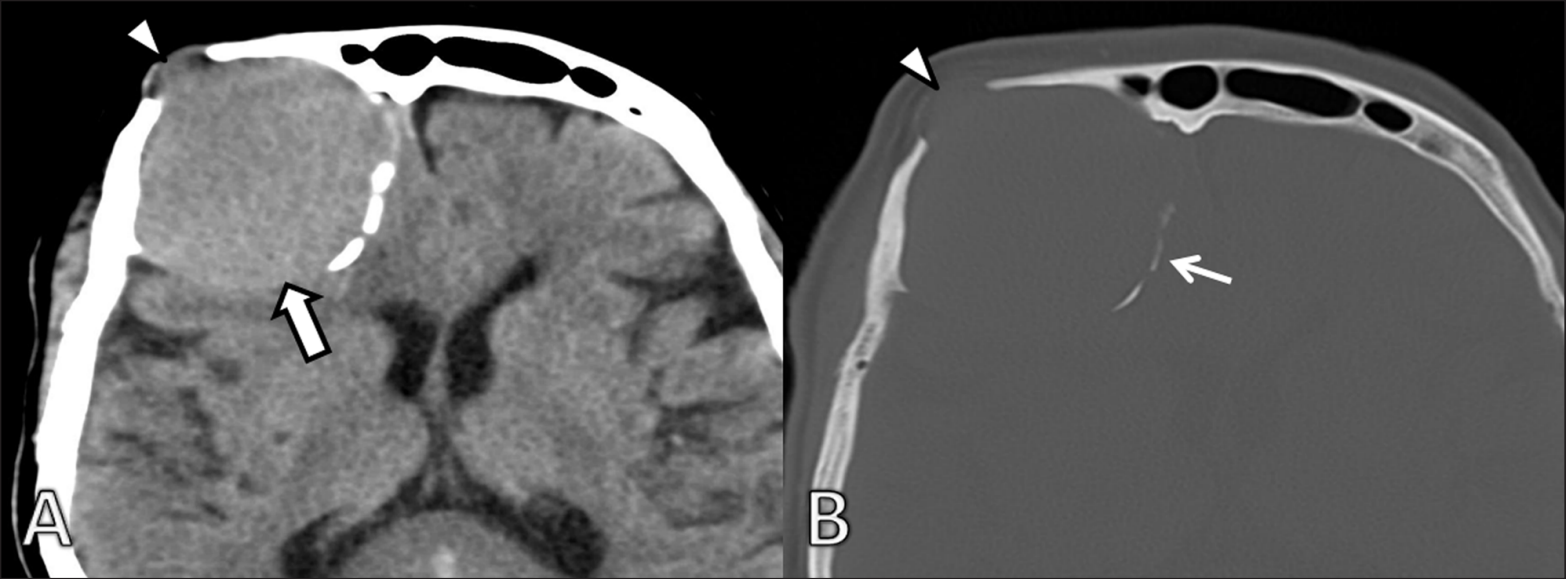
Initial axial non-enhanced CT. **A.** Soft tissue and **B.** bone window show a slightly hyperdense mass to gray matter (arrow) located at the right frontal bone causing scalloping of the internal and external table (arrowhead). There is focal discontinuity of the external and internal table. Remnants of the expanded frontal bone (thin arrow) can be seen medially.

A 72-year-old woman was admitted at the emergency department after a fall. A large bump was noted on her left forehead and subsequently a non contrast-enhanced CT of the head was performed (Figure 1A). The CT scan revealed a large extra-axial slightly hyperdense mass on the right frontal bone. There was expansion of the frontal bone with thinning of the internal and external tables and even some focal cortical discontinuities (Figure 1B).

**Figure 2:**
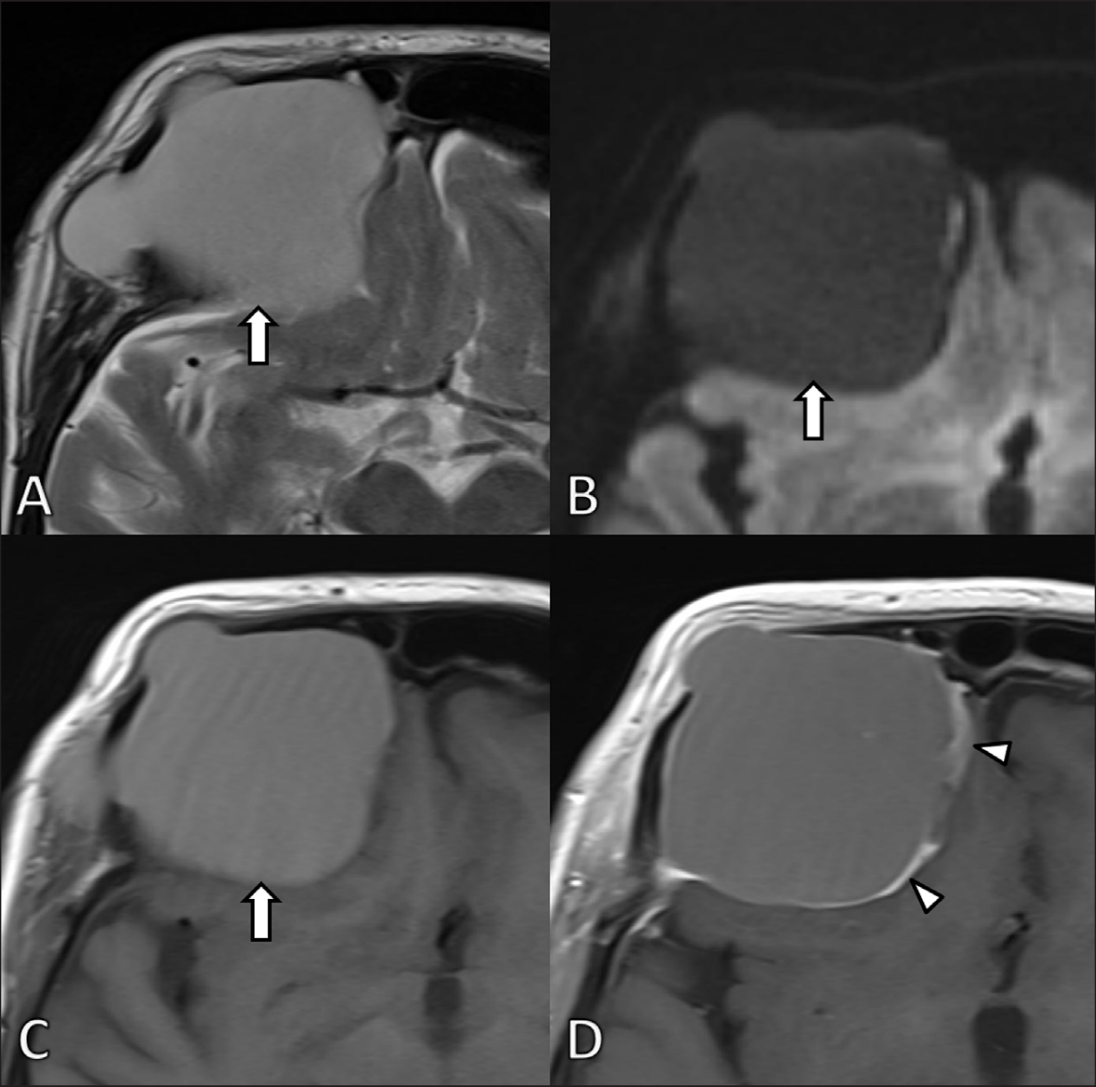
MRI at first admission. **A.** Axial T2-WI. **B.** Diffusion weighted image (b1000) and **C.** T1-WI before and **D.** after gadolinium contrast administration confirm the presence of a well-defined expansile mass (arrow) at the frontal bone. The signal is homogenously hyperintense on T2 and T1-WI images in keeping with high protein content. The lesion did not demonstrate restricted diffusion. After administration of gadolinium contrast the lesion shows subtle peripheral contrast enhancement (arrowheads). The lesion exerts mass effect on the frontal lobe.

**Figure 3:**
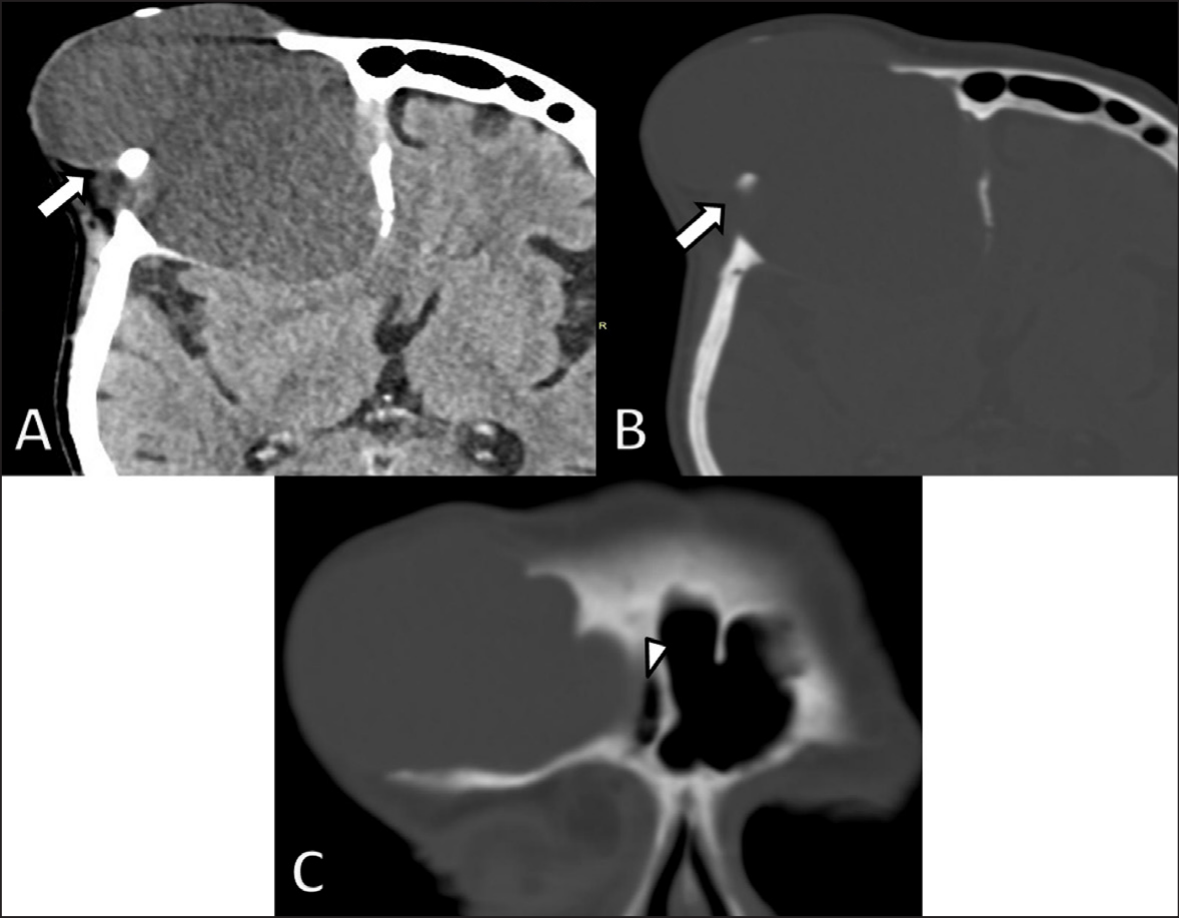
Axial non-enhanced CT images four years later. **A.** Soft tissue and **B.** bone window demonstrate considerable growth of the lesion (arrows) and a decrease in density. There is increased osteolytic destruction of the frontal bone and progressive extra-axial extension into the brain with mass effect on the frontal horn of the lateral ventricle. **C.** Coronal reformatted CT image shows an intimate relationship of the lesion with the right frontal sinus (arrowhead).

**Figure 4:**
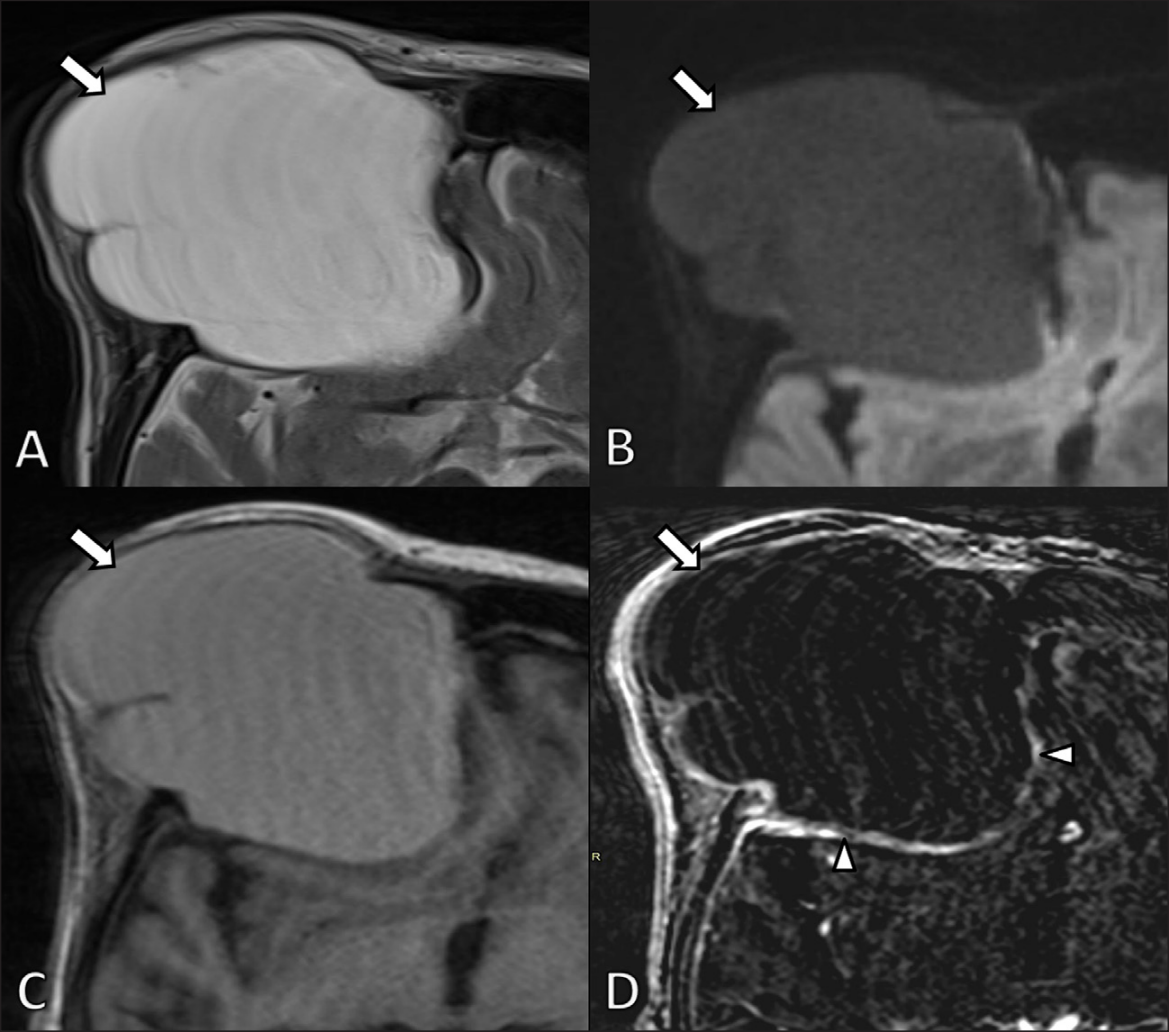
MRI scan 4 years later. **A.** Axial T2-WI. **B.** Diffusion weighted image (b1000) and **C** T1-WI and **D.** subtraction image of T1-WI before and after gadolinium contrast administration. The lesion (arrows) is slightly more hyperintense on T2-WI and slightly less intense on T1-WI compared to the previous examination due to a higher fluid content. There is no diffusion restriction and persistent subtle peripheral contrast enhancement (arrowheads).

Subsequent MRI of the brain (Figure 2) depicted a well-defined expansile mass being slightly hyperintense on T1-weighted images (WI) and markedly hyperintense on T2-WI. A neurosurgical consult was planned but the patient did not show up. The patient was readmitted four years later because of recurrent falls and memory loss. Repeated CT (Figure 3) and MRI (Figure 4) demonstrated progressive expansion of the mass with increased destruction of the frontal bone. Based on the location at the frontal sinus and the imaging features, a presumptive diagnosis of a giant frontal mucocele was made, which was confirmed upon neurosurgical resection.

## Discussion

Paranasal mucoceles are benign, epithelium-lined cysts filled with mucoid material. Mucoceles develop when the sinus ostium is obstructed resulting in progressive accumulation of secretions and epithelial cells in the sinus cavity, with subsequent expansion of the involved sinus [7]. Sinus expansion is a necessary key to finding in the diagnosis of mucocele. In the absence of expansion, the term sinus obstruction should be used [8]. The etiology of obstruction in mucocele is variable and includes inflammation, trauma, and tumor. Predisposing factors are summarized in Table 1 [9].

**Table 1:** Predisposing factors for mucocele formation.


– Chronic sinusitis
– Craniofacial malformations
– Systemic diseases (Cystic fibrosis, Granulomatosis with polyangiitis, …)
– Obstruction by neoplasia
– Surgery
– Facial trauma


The frontal and ethmoid paranasal sinuses are involved in up to 90% of cases. The maxillary sinus is affected less frequently (10%) and the sphenoid sinus only rarely [9]. In children, an unusual variant can involve the nasolacrimal duct [10]. In some cases with extensive osteolytic destruction of the surrounding anatomical structures, the primary site of the mucocele cannot be determined. Young adults (20–40 years) are most commonly affected [7].

Mucoceles that are sufficiently large may exert mass effect on the surrounding anatomic structures. The clinical symptoms vary according to the location [3,11].

A literature search for cases of giant frontal mucoceles yielded 13 relevant articles. Giant mucoceles of other paranasal sinuses are beyond the scope of this article. Cases were included as long as they presented scientific rigour and relevant bibliographic sources. Eight cases were excluded due to a lack of descriptive parameters and/or images of the mucoceles. The remaining five cases are summarized in Table 2.

**Table 2:** Imaging characteristics of giant frontal sinus mucoceles described in previous case reports.

Authors	Size (cm)	General characteristics	Density compared to grey matter on NECT	Enhancement on CECT	MR findings			

					T1-WI SI*	T2-WI SI*	Enhancement	Other

Singh et al. (2019) Case report [5]	8 × 8 × 7	Expansion and thinning of the frontal bone	Hypodense with peripheral calcifications	N/A	Intermediate	High	Peripheral enhancement	No diffusion restriction. Focal organized hemorrhage inside the lesion
Alshoabi, Gameraddin. (2018) Case report [1]	10 × 9		Isodense	Peripheral enhancement	Intermediate	High	N/A	N/A
Carmichael, Kang. (2015) Case report [4]	4 × 6.5		Intermediate density	N/A	N/A	N/A	N/A	N/A
Kawaguchi et al. (2002) Case report [2]	N/A		Isodense with peripheral calcifications	N/A	Intermediate-Low	High	Peripheral enhancement	N/A
Saki et al. (2000) Case report [6]	6.6 × 8.5		Heterogenous iso- and hypodense	N/A	N/A	N/A	N/A	N/A

NECT: Non-enhanced CT, CECT, contrast-enhanced CT, N/A: Data not available. * Signal intensity compared to grey matter.

In the current literature there are no criteria regarding the use of the term “giant” mucocele. Based on the reported dimensions in other case reports, we propose 5 cm as a cut-off value of the lesion size.

CT and MRI are complementary when imaging mucoceles. CT depicts an expansile, homogenous mass with remodeling of the adjacent bone [12]. Occasionally, a mucocele may cause bone destruction simulating an aggressive neoplasm [13].

In the three cases where contrast was administered, subtle peripheral enhancement was seen, similar to characteristics of smaller mucoceles described in the literature. [12].

MRI can be helpful when differentiating mucoceles from other aggressive lesions. Signal intensity on T1-WI is variable (low in case of a low protein content and high in case of a high protein content). All reviewed giant mucoceles were bright on T2-WI, which may be explained by their high fluid content. However, desiccation of the mucocele contents has been described in chronic non-giant cases, resulting in decreased signal intensity on T1- and T2-weighted images [12].

There was no diffusion restriction in our case and the case reported by Singh et al. [5]. Future studies are mandatory to confirm this finding.

Mucoceles should be differentiated from mucus retention cysts. Unlike mucoceles, sinus retention cysts do not result in expansion and thinning of the bony sinus walls [14]. Table 3 summarizes other potential differential diagnoses.

**Table 3:** Differential Diagnosis of mucoceles.


– Mucus retention cyst
– Arachnoid cyst
– (Epi)dermoid cyst
– Malignant neoplasm (both primary and metastatic)


Lastly, surgical excision is the treatment of choice [7].

## Conclusion

Although there is no standard definition regarding the size of a “giant frontal mucocele”, we propose 5 cm as a cut-off, based on the reported dimensions in other cases studies. The typical imaging findings of a giant mucocele consist of a well-defined expansile lesion located at a paranasal sinus and slow progression on serial imaging. Both the density on CT and signal intensity on MRI can be variable depending on the content of the mucocele. The potential role of diffusion-weighted imaging should be elaborated in future reports.
